# Diagnostic reference levels in interventional neuroradiology procedures – a systematic review

**DOI:** 10.1007/s00234-024-03445-5

**Published:** 2024-09-07

**Authors:** Rogério Lopes, Pedro Teles, Joana Santos

**Affiliations:** 1 Unidade Local de Saúde de Gaia e Espinho, University of Porto, Rua Conceição Fernandes s/n, Vila Nova de Gaia, 4434-502 Portugal; 2 Department of Physics and Astronomy, University of Porto, Rua do Campo Alegre, Porto, 4169-007 Portugal; 3 Medical Imaging and Radiotherapy, Instituto Politécnico de Coimbra, ESTESC - Coimbra Health School, Rua 5 de Outubro, S. Martinho do Bispo, Coimbra, 3046-854 Portugal; 4 Centro de Investigação do IPO-Porto, Rua Dr António Bernardino de Almeida, Porto, 4200-072 Portugal

**Keywords:** Diagnostic reference levels, Interventional radiology, Radiation dose, Radiation protection, Neuroradiology, Fluoroscopy

## Abstract

**Introduction:**

The establishment of diagnostic reference levels (DRLs) is challenge for interventional neuroradiology (INR) due to the complexity and variability of its procedures.

**Objective:**

The main objective of this systematic review is to analyse and compare DRLs in fluoroscopy-guided procedures in INR.

**Methods:**

An observational study reporting DRLs in INR procedures, specifically cerebral arteriography, cerebral aneurysm embolisation, cerebral thrombectomy, embolisation of arteriovenous malformations (AVM), arteriovenous fistulas (AVF), retinoblastoma embolisation, and spinal cord arteriography. Comprehensive literature searches for relevant studies published between 2017 and 2023 were conducted using the Scopus, PubMed, and Web of Science databases.

**Results:**

A total of 303 articles were identified through an extensive literature search, with 159 removed due to duplication. The title and abstract of 144 studies were assessed and excluded if they did not meet the inclusion criteria. Thirty-one out of the 144 articles were selected for a thorough full-text screening. Twenty-one articles were included in the review after the complete text screening.

**Conclusion:**

The different conditions of patients undergoing INR procedures pose a barrier to the standardization of DRLs; nevertheless, they are extremely important for monitoring and optimising radiological practices.

## Introduction

The three fundamental principles of radiological protection are justification, optimisation, and dose limits. For optimisation, the International Commission on Radiological Protection (ICRP) recommends the use of diagnostic reference levels (DRLs) [1]. The DRLs are considered vital elements in dose monitoring and are used as enhancers of optimisation and good radiological practices. They represent the 75th percentile (P75) of a dose distribution in a specific radiological procedure [2].

The DRLs are not established as radiation limits, nor are they intended for individual patients; instead, they are extremely important values for comparing local practices with international recommendations or other centers. If the obtained DRLs values exceeds these benchmarks, an investigation should be conducted, and corrective measures should be implemented to optimize radiation dose [1, 3]. The ICRP acknowledges that establishing DRLs for interventional radiology procedures is more complex due to the anatomical and clinical variability of patients and the complexity of the pathologies undergoing treatment [3].

In INR, minimally invasive procedures guided by fluoroscopy are a highly effective treatment option for various neurovascular conditions. However, due to the complexity of pathologies, some procedures may involve high doses of exposure to ionising radiation for both patients and healthcare professionals, increasing the likelihood of deterministic and stochastic effects [4]. Monitoring practices, awareness of radiation effects, advancements in fluoroscopy equipment, and image parameters are fundamental premises to consider in INR procedures aimed at establishing DRLs [4]. However, the exponential increase in endovascular techniques using ionising radiation has not been accompanied by a corresponding increase in awareness and knowledge among healthcare professionals involved [5]. In most cases, there is a significant underestimation of radiation doses for the most common procedures [5].

Radiation protection 175 advocates for comprehensive training in radiological protection to ensure the safety and efficacy of medical procedures involving radiation. This guideline highlights that all team members involved in such procedures must be well-versed in the principles of radiation safety to minimize exposure to both patients and staff [6]. Similarly, the European project Medical Radiation Protection Education and Training (MEDRAPET), which is an European initiative, aims to enhance the quality and consistency of radiological protection education and training across Europe. The project stresses that proper training in radiological protection is essential for maintaining high standards of patient care and occupational safety in medical settings where radiation is used [6].

In summary, education in radiological protection should be considered an essential part of the training process for teams involved in fluoroscopy-guided procedures [5]. It is crucial to implement the principle of optimisation as a promoter of healthcare quality. Therefore, it is important to be familiar with and understand the most relevant dose descriptors, as well as the DRLs calculated in various studies [5].

The air kerma area product (P_KA_), formerly known as dose area product (DAP) or kerma area product (KAP), represents the dose measured by an ionization chamber positioned at the exit of the primary X-ray beam [5, 7, 8]. The air kerma, expressed in Grays (Gy), is multiplied by the cross-sectional area of the primary X-ray beam in cm^2^. The result of this product is expressed in Gy·cm^2^ [5]. Recent equipment internally calculates the value of P_KA_ using exposure factors such as tube voltage (kVp), tube current time product (mAs), beam position in relation to the patient, and field of view (FOV), in addition to the collimation used [5].

The air kerma at the patient entrance reference point (K_a, r_) is calculated as a point located between the X-ray tube and the detector, 15 cm below the isocenter in the direction of the tube’s focal spot, known as the patient entrance reference point. It does not take into account scattered radiation and is expressed in Gy [5]. It is important to emphasize that K_a, r_ does not correspond to the skin dose, does not provide a mapping of the skin dose, nor does it serve as a reference for the peak skin dose (PSD), although it can be used for its estimation [5].

The standard reference for mapping the PSD has been the use of radiochromic films [9]. Recently, new software solutions from different manufacturers have been tested to provide mapping of skin dose, showing promising results compared to radiochromic films [5, 9].

In 2007, the ICRP recommended extending the concept of reference levels to interventional radiology as a means of monitoring patient doses, aiming to prevent unnecessary risks associated with radiation exposure [10, 11]. The determination of DRLs can be conducted through surveys or measurements of radiation doses in hospitals, regions, or a country [11].

In the European Union, the establishment and use of DRLs in each member state have been mandatory since 1997. This requirement was reinforced by the standards set in the new European Directive 2013/59/Euratom, which establishes basic safety standards for protection against the dangers arising from exposure to ionising radiation, published in 2014 [5].

According to ICRP 135, DRLs values should be calculated based on the P75 of the frequency distribution of the analyzed values, including P_KA_, K_a, r_ [5, 11, 12], and fluoroscopy time (FT) [13].

DRLs pose a challenge for INR due to the complexity and variability of procedures [3], clinical indications, and the patient’s weight and height, while not neglecting the crucial importance of image quality. They should be considered dynamic, flexible, and serve as a reference for best practices. However, it is necessary to take into account the patients’ body mass index (BMI), clinical conditions, type of equipment, and the experience of the operators [5]. There are authors who overlook the BMI for head and neck procedures [14].

Cerebrovascular disease has a high incidence rate and is one of the leading causes of death and comorbidity, particularly in cases of stroke [15]. In INR, various deterministic complications arise from the effects of ionising radiation, such as cutaneous erythema, which occurs with a dose limit of 2 Gy, while 7 Gy can cause permanent hair loss [16]. The formation of cataracts is a significant risk, as the patient’s eyes are exposed to radiation throughout the procedure [16]. The dose to the lens of the INR team is also a concern. The ICRP recommends reducing the threshold dose for cataract induction from 2 to 0.5 Gy. As a result, the dose limit for ocular exposure among the team has been reduced from 150 to 20 mSv per year (averaged over a 5-year period) [16].

The main objectives of this systematic review are to analyze the most important dose descriptors in fluoroscopy-guided procedures and to compare the values of DRLs in various existing INR procedures found in recent literature.

## Methodology

We followed the Preferred Reporting Items for Systematic Reviews and Meta-Analyses (PRISMA) guidelines in conducting and presenting this review. The study protocol was registered in the Prospective Register of Systematic Reviews (PROSPERO) under the number CRD42023477160.

The research question for this systematic review is: What are the DRLs in the literature for INR procedures?

Studies published between January 2017 and October 2023 were included, sourced from the Scopus, PubMed, and Web of Science databases. The following keywords were used: “diagnostic reference levels” AND “interventional radiology” OR “radiation dose” OR “radiation protection” OR “neuroradiology” OR “fluoroscopy”. Additional relevant studies were later added to the review.

As a research approach, only English language references in the fields of medicine, healthcare, physics, and astronomy were included, considering original articles published in indexed journals. The in-depth analyzed articles were recorded in an excel file, where various variables considered in the review were detailed, including the author, the type of INR examination, the country, study typology, data collection period, sample size, number of involved centers, study size, fluoroscopy equipment used, the 50th percentile (P50) or median, and the P75 of P_KA_, K_a, r_, and FT. The number of acquired series was not taken into account. Only quality control and regular equipment maintenance are evident in various studies to ensure compliance with all good practice criteria.

The authors reviewed inclusion and exclusion criteria by assessing keywords, reading abstracts, and objectives to determine the inclusion of the articles in the study.

## Results

**Fig. 1:**
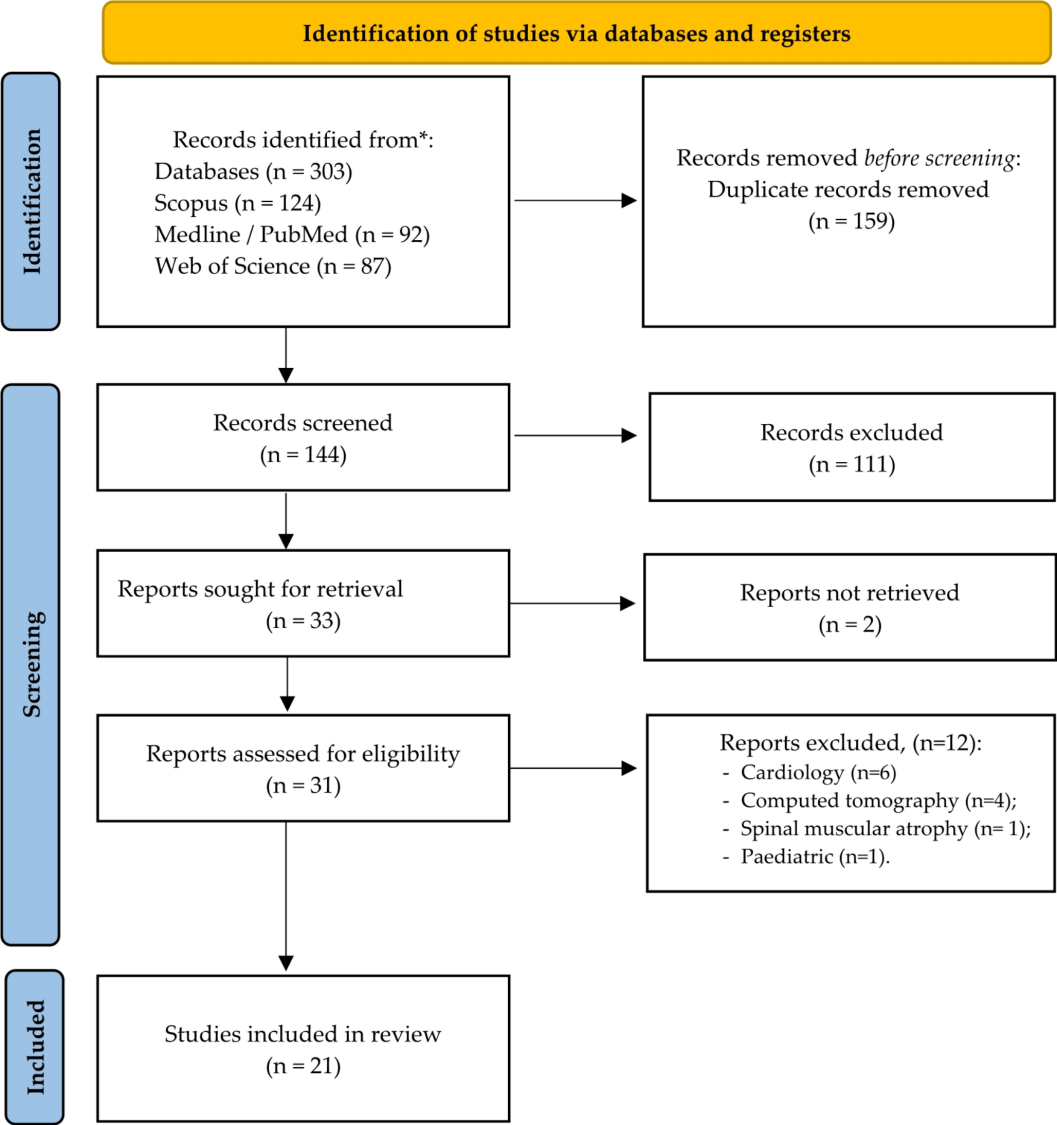
Study selection process according to the PRISMA diagram

As shown in the following flowchart, 303 studies were identified in various databases, for which the authors conducted a thorough analysis and proper screening, leading to the inclusion of 21 studies in the review. Figure 1 depicts the flowchart according to the PRISMA guidelines, summarizing the entire selection process.

### Study results

Initially, 303 articles were identified through an extensive literature search. 159 duplicate articles were removed. The title and abstract of 111 studies were evaluated and excluded for not meeting the inclusion criteria. Subsequently, 33 were selected for full-text screening (studies conducted between 2017 and 2023). 21 articles were included in the review after full-text screening, with 19 originating from the databases and two through the bibliography of analyzed articles conducted in a different time frame from the research.

Characteristics of the included studies:

- Study type;
- Whether DRLs were established based on P75 or P50;
- Indication of the country where the study was conducted;
- Sample size, equipment where data were collected, patients, or hospitals.
- Dose descriptors, P_KA_ presented in Gy·cm^2^, K_a, r_ in mGy, and FT in minutes, and conversion of units when necessary;
- Confirmation that the equipment passed on quality control tests;
- Selected INR examinations, namely diagnostic cerebral arteriography, cerebral aneurysm embolisation, cerebral thrombectomy, embolisation of cerebral AVMs, AVFs, retinoblastoma embolisation, and spinal cord arteriography.

Table 1 presents the variables examined that DRLs for diagnostic cerebral arteriography.

**Table 1:** DRLs studies for diagnostic cerebral arteriography

Cerebral Arteriography													
Authors	Country	Type of study	Data	N	Centres	Equipment	Study	P_KA_ (Gy.cm^2^)		K_a.r_ (mGy)		FT (min)	
								P50	P75	P50	P75	P50	P75
Slave, O. et al. (2023) [1]	South Africa	Retrospective	Jan 2019 -Dec 2019	26	1	Biplane / monoplane	Local	157.5	209.3	598.5	868.5	18.8	28.4
Kanda, R. et al. (2021) [17]	Japan		-	-	Multicentric	-	National	-	89	-	590	-	-
Papanastasiou, E. et al. (2021) [18]	Greece	Prospective	Apr 2019 – Feb 2020	60 (mean 80.5 kg/person)	1	Canon INFX8000 V/GC (Infinix-i Biplane)	Local	50.4	70.2	367.5	494	5.2	9.2
Acton, H. et al. (2018) [16]	Ireland	Retrospective	Nov 2014-Nov 2015	189 (4 cerebral arteries)	1	Axiom Artis dBA	Local	74	96	-	-	-	-
Ihn, Y. et al. (2021) [19]	South Korea	Retrospective	Dec 2020 – Jun 2021	429	22	Biplane (14 Siemens, 7 Philips, and 1 GE)	Regional	70.4	101.6	449.2	711.3	8.6	13.3
Isoardia, P. (2019) [12]	Italy	Retrospective	Jan 2015 -Jan 2019	981	10	Biplane	Local	-	159	-	1401	-	10
Etard, C. (2017) [10]	France	Retrospective	Feb 2016 -Mai 2016	109 (1 art. cerebral)	7	-	National	16.8	26.3	108	218	2.5	3.5
				148 (2 cerebral arteries)	8	-		38.1	71.9	244	462	4.5	6.8
				438 (≥ 3 cerebral arteries)	19	-		65.2	103.2	456	723	7.6	12.3
				695	34	-		45.4	90	312	630	5.7	11
Lee, M. et al. (2019) [11]	Switzerland	Retrospective	2007	-	-	-	-	-	121.5	-	-	-	15
	South Korea	Retrospective	2007–2012	2083 (femoral access)	18 (29 rooms)	-	-	-	188.5	-	-	-	11.4
Tristram. J. et al. (2022) [20]	Germany	Retrospective	Jun 2015 – Apr 2018	161 (1 cerebral artery)	1	Allura Xper FD20 biplane	Local	30.8	61.1	195	340	5.3	13.8
				84 (2 cerebral arteries)	1			48.5	71.4	253	379	6,1	10,4
Vano, E. et al. (2008) [21]	European study (20 countries)	Retrospective	-	72	3 centres in 3 countries	-	European	-	107	-	-	-	12

Table 2 relate studies on DRLs for cerebral aneurysm embolisation.

**Table 2:** Studies on DRLs for cerebral aneurysm embolisation

Cerebral Aneurysm Embolisation													
Authors	Country	Type of study	Data	N	Centres	Equipment	Study	P_KA_ (Gy.cm^2^)		K_a.r_ (mGy)		FT (min)	
								P50	P75	P50	P75	P50	P75
Kanda. R. et al. (2021) [21]	Japan	Retrospective	-	-	Multicentric	-	Nacional	-	210	-	3100	-	-
Schegerer A. et al. (2019) [22]	Germany	Retrospective	2016–2018	-	Multicentric	-	Nacional	121	192	-	-	34	54
Acton. H. et al. (2018) [16]	Ireland	Retrospective	Nov 2014 – Sep 2016	109	1	Axiom Artis dBA	Local	100	123	-	-	-	-
Ihn. Y. et al. (2021) [19]	South Korea	Retrospective	Dez 2020 – Jun 2021	327	22	Biplane (14 S. 7 Philips. and 1 GE machines)	Regional	130.6	199.6	2104.0	3458.7	40.9	57.3
Isoardia, P. et al. (2019) [12]	Italy	Retrospective	Jan 2015 – Jan 2019	489	7 (only a median)	Biplane	Local	164.5	-	2197	-	32.1	-
Etard, C. (2017) [10]	France	Retrospective	Feb 2016 -May 2016	427	19	-	Nacional	130.8	190	1718	2770	37.2	58
Opitz, M. et al. (2023) [23]	Germany	Retrospective	2010–2021	583	1	Allura Xper FD20/10 biplane	Local	157	217	-	-	32.7	-
								125.5 (unruptured)	183 (unruptured)			29.2 (unruptured)	
								187.2 (ruptured)	246 (ruptured)			36.3 (ruptured)	
Lee, M. et al. (2019) [11]	USA	Retrospective	2003–2009	1350	-	-	-	-	360	-	4750	-	90
	Germany	Retrospective	2016–2019	-	-	-	-	-	250	-	-	-	54
	Switzerland	Retrospective	2007	-	-	-	-	-	440	-	-	-	50
	South Korea	Retrospective	2007–2012	320	15 (18 rooms)	-	-	-	383.5	-	-	-	49.6
Tristram, J. et al. (2022) [20]	Germany	Retrospective	Jun 2015 – Apr 2018	129	1	Allura Xper FD20 biplane	Local	132.8	186.8	1397	1906	51.4	70
Miller, D. et al. (2009) [24]	USA	Prospective	1999–2002	134 (57 between 60-80 kg)	7	-	Nacional	-	360	-	4750	-	90

According to the study by Isoardia, P. et al. (2019), they only defined the median of the P_KA_ values in the cerebral aneurysm embolisation.

The studies considered for cerebral thrombectomy are highlighted in Table 3.

**Table 3:** Studies on DRLs for cerebral thrombectomy (recanalization of cerebral arteries)

Cerebral Thrombectomy													
Authors	Country	Type of study	Data	N	Centres	Equipment	Study	P_KA_ (Gy.cm^2^)		K_a, r_ (mGy)		FT (min)	
								P50	P75	P50	P75	P50	P75
Schegerer A. et al. (2019) [22]	Germany	Retrospective	2016–2018	-	Multicentric	-	Nacional	91	158	-	-	21	35
Pace, E. et al. (2020) [25]	Malta	Retrospective	2016–2019	122	1	Philips Allura Clarity FD20/15 biplane	Local	51.4	120.2	-	-	-	-
Acton, H. et al. (2018) [16]	Ireland	Retrospective	Nov 2014 – Nov 2015	10	1	Axiom Artis dBA	Local	87	172	-	-	-	-
Ihn, Y. et al. (2021) [19]	South Korea	Retrospective	Dec 2020 – Jun 2021	326	22	Biplanar (14 S, 7 Philips, and 1 GE machines)	Regional	150.4	225.1	1036.0	1590.0	28.6	44.7
Guenego, A. et al. (2019) [26]	4 (France, Switzerland, USA, and Canada)	Retrospective	Jan 2014 – Mai 2017	576 (biplanar without dose reduction)	5	Biplanar	Local	140	187	970	1210	22	37
				520 (biplanar with dose reduction)		Biplane with dose reduction		91	148	460	730	18	28
Bärenfänger, F. et al. (2023) [27]	Germany	Retrospective	2019–2021	41,538	180	-	-	52.8	140	-	-	-	-
Weyland, C. et al. (2020) [14]	Germany	Retrospective	Jan 2013 – Apr 2018	204 (1 attempt)	1	Artis Zee Biplane and Artis Q	Local	-	107	-	-	-	29
				122 (2 attempt)				-	156	-	-	-	41
				79 (3 attempt)				-	184	-	-	-	45
				59 (4 attempt)				-	244	-	-	-	76
				31 (5 attempt)				-	295	-	-	-	98
				49 (> 5 attempt)				-	333	-	-	-	98
				544 (total) – apenas na circulação anterior				113.7	181.7	-	-	31	53
Lee, M. et al. (2019) [11]	Germany	Retrospective	2016–2019	-	-	-	-	-	180	-	-	-	35
Tristram, J. et al. (2022) [20]	Germany	Retrospective	Jun 2015 – Apr 2018	457	1	Allura Xper FD20 biplane	Local	102.9	151.9	653	1032	25.8	40.3

Tables 4 and 5 display the DRLs results for cerebral AVMs and AVFs, respectively.

**Table 4:** Studies on DRLs for AVMs embolisation

AVM Embolisation													
Authors	Country	Type of study	Data	N	Centres	Equipment	Study	P_KA_ (Gy.cm^2^)		K_a, r_ (mGy)		FT (min)	
								P50	P75	P50	P75	P50	P75
Acton, H. et al. (2018) [16]	Ireland	Retrospective	Nov 2014 -Nov 2015	6	1	Axiom Artis dBA	Local	259	310	-	-	-	-
Ihn, Y. et al. (2021) [19]	South Korea	Retrospective	Dec 2020 – Jun 2021	78	22	Biplane (14 S, 7 Philips, and 1 GE machines)	Regional	264.3	412.3	3073.5	4447.8	63.1	99.3
Etard, C. (2017) [10]	France	Retrospective	Feb 2016 -Mai 2016	239	13	-	Nacional	169.9	285	2019	3230	44.5	68
Lee, M. et al. (2019) [11]	USA	Retrospective	2003–2009	1500	-	-	-	-	550	-	6000	-	135
Miller, D. et al. (2009) [24]	USA	Prospective	1999–2002	148 (69 between 60-80 kg)	7	-	Nacional	-	550	-	6000	-	135

**Table 5:** Studies on DRLs for AVFs.

AVF Embolisation														
Authors	Anatomic region	Country	Type of study	Data	N	Centres	Equipment	Study	P_KA_ (Gy.cm^2^)		K_a, r_ (mGy)		FT (min)	
									P50	P75	P50	P75	P50	P75
Opitz, M. et al. (2022) [4]	Cerebral	Germany	Retrospective	Feb 2010 – Feb 2020	71 (diagnostic)	1	Allura Xper FD20/10 biplane	Local	214.19	256.65	-	-	17.18	-
					11 (treatment)				369.79	507.33	-	-	58.57	-
	Spinal				58 (diagnostic)				210.57	396.39	-	-	25.33	-
					24 (treatment)				275.98	482.72	-	-	35.45	-
Lee, M. et al. (2019) [11]	Spinal	USA	Retrospective	2003–2009	1500	-	-	-	-	950	-	8000	-	130

In Table 6, we present the studies on DRLs for retinoblastoma embolisation, and in Table 7, the results of spinal cord angiography.

**Table 6:** Retinoblastoma embolisation

Retinoblatoma Embolisation															
Authors	Country	Type of study	Data	N		Centres	Equipment	Study	P_KA_ (Gy.cm^2^)			K_a, r_ (mGy)		FT (min)	
									Mean	P50	P75	P50	P75	Mean	P50
Opitz et al. (2021) [2]	Germany	Retrospective	Jan 2010 – Mar 2020	248	0 (1–3 months)	1	Jan 2010 - Nov 2018 - Allura Xper FD20/10 biplane Nov 2018 - Mar 2020 - Siemens Artis Q biplane	Local	-	-	-	-	-	-	-
					85 (4–12 months)				-	2.9	3.9	-	-	-	7.41
					157 (13–72 months)				-	4.3	7.0	-	-	-	7.52
					4 (73 months – 10 years)				-	10.7	14.5	-	-	-	6.20
					2 (> 10 years)				-	8.8	-	-	-	-	4.47
Qureshi, A.M. et al. (2019) [28]	UK	Retrospective	Jan 2013 - Dec 2017	178 (48 with retinoblastoma)		1	Siemens Artis Zee biplane	Local	11.7± 9.7	-	-	-	-	13.5 ± 13	-
Obesso, A. et al. (2019) [29]	Spain	Retrospective	Mar 2016 – Feb 2018	16 (35 procedures for ages 0–5 years)		1	Philips Allura FD20 monoplane system and a Philips Allura Clarity FD20/15 biplane system	Local	-	16	-	130	-	-	-

**Table 7:** Studies on DRLs for spinal cord angiography

Spinal Angiography													
Authors	Country	Type of study	Data	N	Centres	Equipment	Study	P_ka_ (Gy.cm^2^)		K_a, r_ (mGy)		FT (min)	
								P50	P75	P50	P75	P50	P75
Etard, C. (2017) [10]	France	Retrospective	Feb 2016 -May 2016	123	7	-	Nacional	88	185	639	1420	15.1	26
Tristram, J. et al. (2022) [20]	Germany	Retrospective	Jun 2015 – Apr 2018	16	1	Allura Xper FD20 biplane	Local	417.6	530.8	2848	3165	24.3	33.6

## Discussion

The P_KA_ and K_a, r_ reported by fluoroscopy equipment are used for optimisation purposes and are employed in defining the DRLs [5]. The comparison of results across different analyzed procedures highlights a significant variability in values. The most commonly used metric is P_KA_, except in the USA, where the K_a, r_ value is a parameter required by the Food and Drug Administration [10].

Comparing these studies is challenging due to inconsistencies in describing the performed procedures and a lack of information on complexity levels during the interventions. Some articles provide only mean P_KA_ values instead of median and quartile values or interquartile ranges (P75 for DRLs estimation). This lack of consistent information on the type of procedure and the absence of specification of complexity levels result in a broad range of dose and FT values found in publications [30]. The practical approach would be to collect a reasonable set (a minimum of 20 to 30 procedures) of dosimetric data for well-defined clinical indications and calculate the 3rd quartile values [3, 30, 31].

The complexity of cerebrovascular anatomy and the procedures themselves can lead to high radiation doses. According to ICRP Publication 135, the use of various radiation dose metrics is recommended for establishing DRLs in fluoroscopy-guided interventions [3]. The radiation dose in INR procedures is primarily influenced by the complexity of the procedure rather than the patient’s BMI [4, 32]. The establishment of DRLs is also defined by the European Union Directive 2013/59/Euratom and is one of the requirements of the International Basic Safety Standards published by the International Atomic Energy Agency [31].

According to Radiation Protection 195, European Study on Clinical Diagnostic Reference Levels for X-ray Medical Imaging, the DRLs values are based on local studies with reference to clinical indication and literature review however this report only propose body procedures for interventional radiology as arterial occlusive disease of iliac arteries, transarterial chemoembolization, arterial occlusive disease of femoropopliteal arteries and biliary drainage [30].

A RP 195 reviews the DRLs in various European Union countries for NRI procedures. Belgium presents 175 Gy.cm^2^ for monopolar equipment and 240 Gy.cm^2^ for biplanar. France shows 190 Gy.cm^2^ with 58 min of FT. In turn, Germany presents 180 Gy.cm^2^ for aspiration thrombectomy and 250 Gy.cm^2^ for cerebral aneurysm embolisation with coils. Ireland demonstrates 62 Gy.cm^2^ in studies, and Switzerland, 350 Gy.cm^2^ of P_KA_ with 50 min of FT [30].

According to Slave, O. et al., cerebral angiography exceeds the DRLs values when compared to other published studies and presents a small sample size. Therefore, this procedure needs to be reviewed and optimized according to the recommendations of ICRP 135 [1, 3].

Based on Acton, et al., the number of cerebral thrombectomies and AVMs interventions performed was low, and the P75 values of 172 Gy.cm^2^ for stroke intervention procedures and 310 Gy.cm^2^ for AVM embolisation provide a preliminary guide; however, they cannot be defined as DRLs [16].

In the study by Iln, Y. et al., P_KA_ did not show significant differences regarding the occlusion site or success in recanalization in cerebral thrombectomies. However, the number of attempts needed to remove a clot is known to reflect the complexity of the procedure. This parameter has proven to be the most significant factor in increasing patient dose, as corroborated by the study of Weyland, C. et al. [14, 19].

It is known that NRI procedures can vary considerably in the complexity of similar procedures due to the type and extent of pathology or the patient’s clinical condition during the procedure. It is necessary to define typical dose values and DRLs taking into account not only the clinical indication but also the level of procedure complexity [5]. According to Table 2 and graph 2, P_KA_ values for cerebral aneurysm embolisation vary from 123 Gy.cm^2^ to 440 Gy.cm^2^. The same trend is observed in other studies under analysis.

According to Curtis et al., embolisation of the vein of Galen in a fetus at 10 weeks resulted in a PSD exposure of 480 mGy [33]. Orbach et al. examined a total of 175 pediatric neurointerventions between September 2006 and July 2010, and an additional 180 cases between July 2010 and June 2012. The examined NRI procedures encompassed various cerebrovascular pathologies, such as AVMs, pial fistulas, aneurysms, dural fistulas, and extracranial AVMs or AVFs, present in all age groups. Vein of Galen malformations were exclusively present in the age groups < 1 year and 1 to 2 years [34]. In the same study, the PSD reached 372.9 mGy in those under 1 year and 443.5 mGy in the age group of 1 to 2 years [34].

The radiation exposure in comparatively complex interventions, such as AVMs embolisation, is similar to FAVs embolisation. It may be helpful to determine general DRLs for both entities together [4].

Establishing DRLs for NRI procedures is a challenging task due to the significant variability in FT and the number of acquired images, resulting in a wide distribution of doses in patients, as evidenced by the results of this review. Additionally, the types of equipment with different acquisition technologies, institutional protocols, and operator preferences for procedures can make it difficult to compare radiation doses between hospitals [19]. Despite observing very different DRLs values for the same diagnostic procedures, this issue can be addressed by standardizing protocols and appropriately utilizing dose reduction tools. Furthermore, and more importantly, it is necessary to implement policies for education, training and fostering a culture of radiological protection among healthcare professionals [5].

The limitation verified were a small number of studies conducted, especially in the embolisation of retinoblastomas and spinal cord arteriography, which prevents comparisons with other local DRLs. In the case of AVMs, MAVs, and there is also some shortage of studies in this clinical condition. Parameters such as BMI and procedure complexity were not included in the majority of studies. The BMI of patients is an influencing factor in radiation exposure, with greater relevance in diagnostic and interventional imaging procedures in the thoracic and abdominal regions and less influence in head and neck procedures [14].

## Conclusions

Interventional neuroradiology procedures exhibit a wide variety and complexity. All studies (100%) of cerebral arteriography and cerebral thrombectomy reported the P75 value of P_KA_, and 70% and 60% of the studies reported the P75 values of K_a, r_, respectively. In studies of aneurysm embolisation, 90% reported the P75 values of P_KA_, and only 50% reported the values of K_a, r_. P_KA_ is the predominant metric in common use in DRLs.

The radiation dose can vary depending on their complexity; however, it is the responsibility of healthcare professionals, including physicians and radiographers, to monitor their own practice and reduce radiation exposure according to the ALARA (As Low as Reasonably Achievable) principle. The ongoing evolution of practices and technologies involved requires regular updates to DRLs.
